# High-Throughput Phenotyping of Bioethanol Potential in Cereals Using UAV-Based Multi-Spectral Imagery

**DOI:** 10.3389/fpls.2019.00948

**Published:** 2019-07-23

**Authors:** Francisco J. Ostos-Garrido, Ana I. de Castro, Jorge Torres-Sánchez, Fernando Pistón, José M. Peña

**Affiliations:** ^1^Institute for Sustainable Agriculture, Spanish National Research Council (CSIC), Córdoba, Spain; ^2^Institute of Agricultural Sciences, Spanish National Research Council (CSIC), Madrid, Spain

**Keywords:** remote sensing, unmanned aerial vehicle, vegetation index, biomass, sugar release, theoretical ethanol yield, breeding program

## Abstract

Bioethanol production obtained from cereal straw has aroused great interest in recent years, which has led to the development of breeding programs to improve the quality of lignocellulosic material in terms of the biomass and sugar content. This process requires the analysis of genotype–phenotype relationships, and although genotyping tools are very advanced, phenotypic tools are not usually capable of satisfying the massive evaluation that is required to identify potential characters for bioethanol production in field trials. However, unmanned aerial vehicle (UAV) platforms have demonstrated their capacity for efficient and non-destructive acquisition of crop data with an application in high-throughput phenotyping. This work shows the first evaluation of UAV-based multi-spectral images for estimating bioethanol-related variables (total biomass dry weight, sugar release, and theoretical ethanol yield) of several accessions of wheat, barley, and triticale (234 cereal plots). The full procedure involved several stages: (1) the acquisition of multi-temporal UAV images by a six-band camera along different crop phenology stages (94, 104, 119, 130, 143, 161, and 175 days after sowing), (2) the generation of ortho-mosaicked images of the full field experiment, (3) the image analysis with an object-based (OBIA) algorithm and the calculation of vegetation indices (VIs), (4) the statistical analysis of spectral data and bioethanol-related variables to predict a UAV-based ranking of cereal accessions in terms of theoretical ethanol yield. The UAV-based system captured the high variability observed in the field trials over time. Three VIs created with visible wavebands and four VIs that incorporated the near-infrared (NIR) waveband were studied, obtaining that the NIR-based VIs were the best at estimating the crop biomass, while the visible-based VIs were suitable for estimating crop sugar release. The temporal factor was very helpful in achieving better estimations. The results that were obtained from single dates [i.e., temporal scenario 1 (TS-1)] were always less accurate for estimating the sugar release than those obtained in TS-2 (i.e., averaging the values of each VI obtained during plant anthesis) and less accurate for estimating the crop biomass and theoretical ethanol yield than those obtained in TS-3 (i.e., averaging the values of each VI obtained during full crop development). The highest correlation to theoretical ethanol yield was obtained with the normalized difference vegetation index (*R*^2^ = 0.66), which allowed to rank the cereal accessions in terms of potential for bioethanol production.

## Introduction

Currently, there is a renewed interest in biomass recovery for energy consumption because biomass is a renewable and carbon neutral source of energy (Perea-Moreno et al., 2019). Two types of biofuels can be distinguished according to the different feedstock types. The first generation liquid biofuel is produced from cereals, sugar crops, and oilseeds, and the second-generation liquid biofuel is produced from lignocellulosic feedstock (Mittal and Decker, 2013; Kang et al., 2014). Between the two types, second-generation biofuel is a more sustainable option because it is not in direct competition with the food supply and, consequently, it does not increase food prices. Additionally, it produces lower greenhouse gas emissions and better water and land uses (Sims et al., 2010). The process of biofuel production could be improved in terms of productivity, efficiency and cost reduction by using two powerful tools, classical breeding and biotechnology, and by analyzing the genotype–phenotype relationships in both cases.

Genotyping tools have been investigated in deep over the last 20 years and have led to a better understanding of the plant genome through DNA sequencing and molecular technologies. In contrast, that investigation has not happened with phenotyping tools, which usually have been unable to satisfy the greatest number of technical requirements non-destructively with high performance and speed at a low price (Araus and Cairns, 2014). However, in recent years, new high-throughput phenotyping platforms are undergoing a rapid evolution that could significantly improve the understanding about the association between genes and phenotypes (White et al., 2012; Cobb et al., 2013; Dhondt et al., 2013). These platforms for phenotyping are capable of generating large quantities of data quickly and give the opportunity to evaluate plants in actual field conditions. Several high-throughput platforms had been developed for non-destructive plant data collection, as examples, tractor-mounted platforms (Busemeyer et al., 2013; Walter et al., 2019), cable-driven platform (White and Bostelman, 2011), aerial vehicles (Shi et al., 2016; Tattaris et al., 2016), and portable or pushed platforms (Jay et al., 2015; Bai et al., 2016; Crain et al., 2016), among others (Araus et al., 2018; Rouphael et al., 2018). In the specific case of cereals, some of these high-throughput sensor-based techniques have been applied, e.g., for assessing salt-tolerance in *Triticum* (Rajendran et al., 2009), drought-tolerance in barley (Hartmann et al., 2011) and maize (Winterhalter et al., 2011), biomass and/or plant height in maize (Han et al., 2019), barley (Bendig et al., 2014), triticale (Alheit et al., 2014), wheat (Yue et al., 2017; Hassan et al., 2019) and sorghum (Watanabe et al., 2017), growth status in wheat (Du and Noguchi, 2017), wheat spike (Zhou et al., 2018), and seedling emergence and spring stand in winter wheat (Sankaran et al., 2015a). However, to our knowledge, there are no works in which image-based technologies have been used to evaluate the potential for bioethanol production from cereals grown in field conditions. In this specific case, the cultivation of cereals with the dual fitness of providing the grains for food and the crop residues for bioethanol production has been the subject of many investigations, in which the main interest lies in obtaining organic residues with a cell wall that is easily degradable by enzymes but do not compromise grain yield (Jensen et al., 2011; Lindedam et al., 2012).

In recent years, unmanned aerial vehicles (UAVs) have become a relevant tool for phenotyping because of their advantages over other platforms (Yang et al., 2017). UAVs are a low-cost and reliable method for taking remote images by using global positioning and inertial navigation systems, which allows for frequent field observations to capture the variation of plant traits over time. Furthermore, the capacities of the UAV to use a wide range of sensors and to operate at a low flight altitude provide high-resolution spatial and spectral information on the studied plants (Peña et al., 2015). Based on the knowledge of phenotypes related to the bioethanol potential and on the capability of the UAVs to collect high-resolution images in field trials, a UAV-based phenotyping system was developed and tested on a multi-temporal field experiment composed of 66 genotypes belonging to several species of cereal crops. First, this work describes the full protocol to collect remote images with a multi-spectral camera and to analyze the images by using a customized object-based image analysis (OBIA) algorithm. The OBIA partitions the image in spatially and spectrally homogenous objects following a segmentation process and then combine several features of spectral information, location, proximity and hierarchy of the segmented objects to analyze the vegetation fraction (Torres-Sánchez et al., 2014). The use of OBIA methodology drastically increase the success of vegetation classification and facilitate multi-temporal analysis of vegetation blocks. Then, a comparison of multi-temporal UAV-based data and on-ground measurements of the crop trials allowed the correlation between image spectral information in the visible and near-infrared spectrum regions (by focusing to specific vegetation indices) and three primary variables related to bioethanol potential (i.e., total biomass dry weight, sugar release, and theoretical ethanol yield) as affected by species and several temporal scenarios (TSs) to be determined. The final target of the UAV-based phenotyping system was to provide a ranking of accessions in terms of the bioethanol potential with value for facilitating the decision making process in the context of plant breeding programs.

## Materials and Methods

### Field Trial and Plant Material

A field trial with 66 accessions belonging to the species *Hordeum vulgare* (barley, 21 accessions), *Triticum aestivum* (bread wheat, 24 accessions), *Triticum durum* (durum wheat, 11 accessions) and *x Triticosecale* (triticale, a hybrid of wheat and rye, 10 accessions) was established at the experimental station of the Institute for Sustainable Agriculture Center in Cordoba, Spain (Table 1).

**TABLE 1:** Plant material used in this investigation.

**Species**	**ID**	**Accession name^a^**	**Accession number**
***T. aestivum***	Anza	Anza ^*^	NA
	BW	BobWhite ^*^	NA
	Peri	Perico ^*^	NA
	TP2	UC1110 ^∗∗^	GSTR 13501
	TP3	OS9A ^∗∗^	PI658243
	TP4	QCB 36 ^∗∗^	PI658244
	TP5	Opata 85 ^∗∗^	PI591776
	TP6	Cayuga ^∗∗^	PI595848
	TP7	Caledonia ^∗∗^	PI610188
	TP8	CIGM90.248 ^∗∗^	PI610750
	TP10	P91193 ^∗∗^	GSTR 10001
	TP11	P92201 ^∗∗^	GSTR 10002
	TP16	TAM107-R7 ^∗∗^	GSTR 11601
	TP17	SS550 ^∗∗^	GSTR 12501
	TP21	M6 ^∗∗^	PI83534
	TP22	Kanqueen ^∗∗^	PI401539
	TP24	Avocet ^∗∗^	PI464644
	TP25	Penawawa ^∗∗^	PI495916
	TP27	Renan ^∗∗^	PI564569
	TP28	Excalibur ^∗∗^	PI572701
	TP29	McNeal ^∗∗^	PI574642
	TP30	Thatcher ^∗∗^	CItr 10003
	TP31	Jaypee ^∗∗^	PI592760
	TP32	USG 3209 ^∗∗^	PI617055
	TP33	Caledonia ^∗∗^	PI610188
	TP34	Cayuga ^∗∗^	PI595848
***T. durum***	TP1	IDO444 ^∗∗^	GSTR 12902
	TP9	UC1113 Yr36 Gpc-B1 ^∗∗^	PI638741
	TP12	Grandin^*^5/ND614-A ^∗∗^	GSTR 10401
	TP13	NY18/Clark’s Cream 40-1 ^∗∗^	GSTR 10402
	TP14	Jupateco 73S ^∗∗^	GSTR 10501
	TP15	CO940610 ^∗∗^	GSTR 10702
	TP18	Amadina ^∗∗^	GSTR 12701
	TP19	Weebill 1 ^∗∗^	GSTR 10502
	TP20	Rugby ^∗∗^	CItr 17284
	TP23	Avalon ^∗∗^	PI446910
	TP26	Rio Blanco ^∗∗^	PI531244
***H. vulgare***	CP1	Vada ^∗∗^	PI280422
	CP2	Clipper ^∗∗^	PI349366
	CP3	Ko A ^∗∗^	PI383935
	CP4	Igri ^∗∗^	PI406263
	CP5	Mokusekko 3 ^∗∗^	PI420938
	CP6	Dicktoo ^∗∗^	CIho 5529
	CP7	L94 ^∗∗^	CIho 11797
	CP8	Fredrickson ^∗∗^	CIho 13647
	CP9	Steptoe ^∗∗^	CIho 15229
	CP10	Morex ^∗∗^	Ciho 15773
	CP11	Lina ^∗∗^	PI584808
	CP12	Apex ^∗∗^	PI600966
	CP13	OWB dominant ^∗∗^	GSHO3450
	CP14	OWB recessive ^∗∗^	GSHO3451
	CP16	Golden Promise ^∗∗^	PI467829
	CP17	Cebada Capa ^∗∗^	PI539113
	CP18	Stander ^∗∗^	PI564743
	CP19	Franklin ^∗∗^	PI373729
	CP20	Franka ^∗∗^	PI574293
	CP21	Azumamugi ^∗∗∗^	J698
	CP22	Kanto Nakate Gold ^∗∗∗^	J518
***x Triticosecale***	TS43	Rahum ^∗∗^	PI422269
	TS45	Zebra ^∗∗^	PI429031
	TS51	Kramer ^∗∗^	PI476216
	TS53	Currency ^∗∗^	PI483066
	TS58	Wapiti ^∗∗^	PI511870
	TS61	Yoreme Tehuacan 75 ^∗∗^	PI519876
	TS67	Navojoa ^∗∗^	PI520421
	TS75	Drira ^∗∗^	PI520478
	TS78	Juanillo 95 ^∗∗^	PI520488
	TS97	Armadillo 130 ^∗∗^	PI583701

^*a*^Plant material availability: ^*^IAS-CSIC; ^∗∗^USDA-ARS, National Small Grain Germplasm Research Facility, Aberdeen, ID, United States; ^∗∗∗^Barley and Wild Plant Resource Center, Institute of Plant Science and Resources, Okayama University, Okayama, Japan.

**FIGURE 1:**
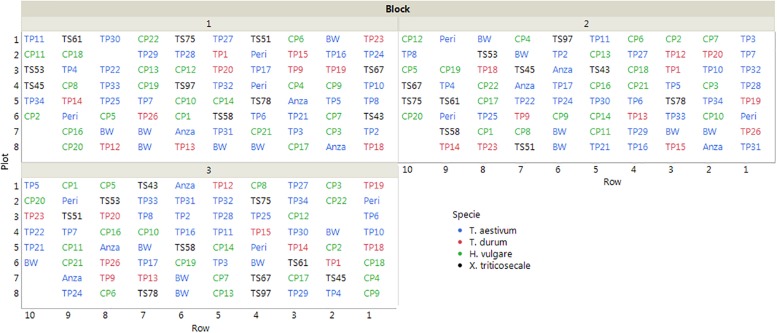
Layout of the field trial (plot IDs correspond to the accessions listed in Table 1). The empty plots were not considered in the evaluation due to errors committed in plant identification.

The cereals were sown on 15 November, 2013 following a completely randomized block design with three replications. Each block was counted in 78 plots that were distributed in 10 rows, with an inter-plot distance of 30 cm and an inter-row distance of 50 cm. Each plot included four plants at approximately 15 cm apart (Figure 1). The cereals were under an irrigation localized system, and the experiment was covered with netting to protect of insects and birds during the growing months.

The Cayuga and Caledonia accessions were planted in duplicate because we had two replicates with different acquisition dates. Plant material was obtained from the USDA-ARS National Small Grain Collection^1^ or from the Barley and Wild Plant Resource Center at Okayama University^2^. When available, the accessions used as parental lines in the cartographic populations were selected according to a double criterion: (1) to allow the identification of suitable cartographic populations to study the genetic basis of saccharification, and (2) to have a fair representation of the available variability in each species, since parental lines are normally selected to be as divergent as possible.

### UAV-Based Phenotyping System

**FIGURE 2:**
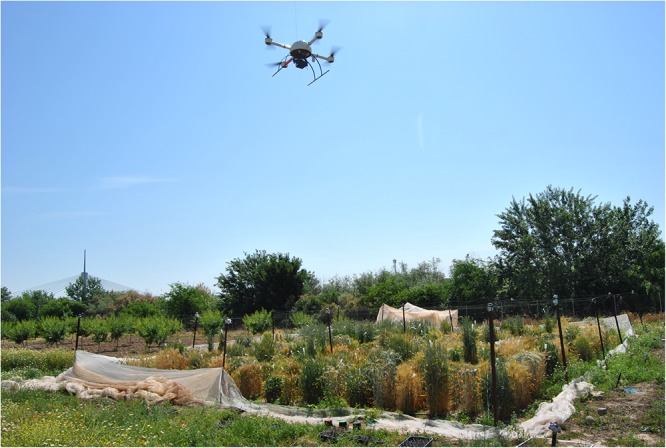
The UAV with the multi-spectral camera flying toward the experimental field on the 7th date (175 DAS).

A quadrocopter UAV model md4-1000 (microdrones GmbH, Siegen, Germany) was used to collect the multi-temporal set of aerial images (Figure 2). The whole system consists of the vehicle, the radio control transmitter, a ground station with software for mission planning and flight control, a telemetry system, and a camera or sensor embedded in the UAV. In this experiment, a six-band Tetracam camera, model mini-MCA-6 (Tetracam, Inc., Chatsworth, CA, United States) was used to collect the multi-spectral images. This camera collected six individual images at B (450 nm), G (530 nm), R (670 mm), R edge (700 nm), and near-infrared (NIR, 740 and 780 nm) by using its user configurable bandpass filters (Andover Corporation, Salem, NH, United States) of 10-nm full-width at half-maximum. These bandwidth filters were selected across the visible and NIR regions with regard to well-known biophysical indices that were developed for vegetation monitoring (Kelcey and Lucieer, 2012).

**FIGURE 3:**
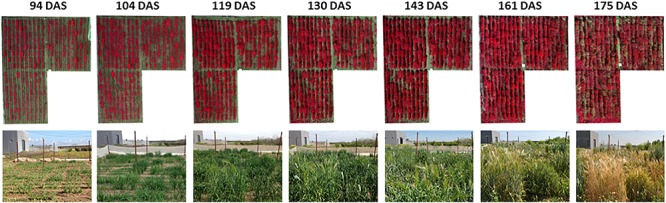
UAV-based ortho-mosaicked images in color-infrared of the experimental field over time. DAS, days after sowing.

The UAV system collected the remote images of the experimental field on seven different dates: (1) 17 February [94 days after sowing (DAS)], (2) 27 February (104 DAS), (3) 14 March (119 DAS), (4) 25 March (130 DAS), (5) 7 April (143 DAS), (6) 25 April (161 DAS), and (7) 9 May (175 DAS). The UAV route was configured to fly at 3 m/s speed and at 10 m above ground level, and to take down-facing photos at 1 s interval with side overlap of 60% and forward overlap of 90%. At this flight altitude, the spatial resolution of the UAV images was 5.41 mm/pixel of ground sampling distance. The set of multi-spectral images was first processed with the PixelWrench2 software (Tetracam, Chatsworth, CA, United States) and then with the Agisoft PhotoScan Professional software (Agisoft LLC, Saint Petersburg, Russia). PixelWrench2 was supplied with the camera and was used to automatically correct the vignette effect, align the six images taken on each camera shot and to create multiband images, as explained in Tetracam (2019). Image alignment consisted on match the individual images from the five slave channels to the image from the master channel by applying the band-to-band registration file that contains information about the translation, rotation and scaling between the six images. Next, a photogrammetric process was performed with Agisoft PhotoScan to generate the ortho-mosaicked images of the entire experimental field by following three consecutive phases: (1) aerial triangulation; (2) building field geometry, and (3) generation of the orthomosaics (Figure 3). This process was automatically performed by the software except for the manual assignment of absolute coordinates to the ortho-mosaicked images from a few ground control points located at the edges of the study field. Readers can found detailed information on these processes in previous investigations (Torres-Sánchez et al., 2013; Mesas-Carrascosa et al., 2017).

The ortho-mosaicked images enabled visual identification of each one of the 234 trial plots, which were manually defined over the image and saved as a vector file. Then, a customized algorithm was created with the eCognition Developer software (Trimble GeoS-patial, Munich, Germany) to analyze the images of each studied date by using an object-based approach after image segmentation (Blaschke et al., 2014). The algorithm was specifically programmed to run in a fully automatic manner without the need for user intervention, and with the ability to sequentially discriminate the vegetation fraction of every trial plot over time by applying the Otsu thresholding method described in Torres-Sánchez et al. (2015). Once the crop objects were classified in each plot, the algorithm computed the central coordinates and the relative position of every plot within the experimental design (row, order in the row and block), the crop spectral values from the multi-spectral camera and a list of crop-related vegetation indices (VIs), which were grouped into those computed from bands in the visible spectrum region (referred to as visible-based VIs) and those that included the NIR band (referred to as NIR-based VIs) (Table 2). These VIs have been commonly used to monitor diverse physiological and phenological crop characteristics that may be relevant in the context of this investigation, such as leaf pigment (e.g., chlorophyll, carotenoid, and anthocyanin), foliage and canopy structure, biomass, non-photosynthetic components (e.g., lignin, cellulose, and starch, etc.) (Asner, 1998; Yang et al., 2017). Finally, the customized algorithm automatically exported all the trial plot data as a table file (e.g., CSV or ASCII format) for further descriptive and statistical analysis.

**TABLE 2:** Crop-based vegetation indices computed in every trial plot.

**Spectral region/vegetation index (VI)**	Equation^*^	**References**
**Visible**		
Excess Green (ExG)	2^*^G−R_1_−B	Woebbecke et al., 1995
Green VI (VIgreen)	(G−R_1_)/(G+ R_1_)	Gitelson et al., 2002
Triangular Chlorophyll Index (TCI)	1.2^*^(R_2_−G)−1.5^*^(R_1_−G)^*^(R_2_/R_1_)^1/2^]	Haboudane et al., 2008
**Visible and NIR**		
Normalized Difference VI (NDVI)	(NIR_1_−R_2_)/(NIR_1_+R_2_)	Rouse et al., 1973
Green NDVI (GNDVI)	(NIR_2_−G)/(NIR_2_+G)	Gitelson and Merzlyak, 1996
Modified Chlorophyll Absorption in Reflectance Index (MCARI)	10.1.1.1 [(NIR_1_−R_2_) −0.2^*^(NIR_1_−G)]^*^(NIR_1_/R_2_)	Daughtry et al., 2000
Modified Simple Ratio (MSR)	[(NIR_1_/R_2_) −1]/[(NIR_1_/R_2_)^1/2^+1]	Chen, 1996

^*^Reflectance (%) at central wavelengths: B = 480 nm (blue region); G = 530 nm (green region); R_1_ = 670 nm (red region); R_2_ = 700 nm (Red-edge region); NIR_1_ = 740 nm (NIR region); NIR_2_ = 780 nm (NIR region).

### Manual Measurements of Plant Phenotypic Data

To evaluate the UAV-based assessments, the total biomass dry weight (kg/m^2^) and sugar release (ul/mg), which are two crop variables that are particularly related to bioethanol potential from lignocellulosic biomass, were determined in each trial plot after harvest. The biomass was measured as weight for complete plant (spike, stem, and leaves) and the sugar release was obtained by using a suitable method of saccharification. Assays to determine saccharification involved three main steps: pretreatment, hydrolysis and sugar detection. First, 20 mg of ground straw were loaded into 2 mL screw-cap tubes. In the pretreatment solution a volume of 1.5 mL of NaOH (6.25 mM) was used and incubated at 90°C for 3 h in a water bath, and it was then cooled on ice. Enzymatic hydrolysis was performed using 0.05 μL of enzyme cocktail with a 4:1 ratio of Celluclast – Novozyme 188 (Novozymes, Bagsværd, Denmark) for 1 straw mg dry weight (dw). Hydrolysis was performed for 20 h with constant shaking at 50°C in a 0.5M sodium citrate buffer at pH 4.5. The determination of sugars released after hydrolysis was carried out using the glucose oxidase/peroxidase (GOPOD) assay kit (Megazyme International Ireland, Bray, Ireland). The assay volumes were reduced to allow the procedure to be performed in 96-well ELISA plates. In every plate, we included solution blanks, enzyme blanks, glucose standards used in the calibration curve as an internal control for the reaction, and eight technical repetitions that left 76 wells free for the samples. These eight samples were formed by eight different genotypes that were randomly selected, except for Anza, Bobwhite, and Perico that were chosen because their glucose releases were previously known. Determination was performed using 8 μL of the digestion reaction mixture and 240 μL GOPOD assay reagent followed by incubation at 50°C for 20 min. The glucose yield was analyzed using 96 well plates. The absorbance readings were determined at 490 nm in a BioTek ELx800 Absorbance Microplate Reader (BioTek Instruments, Inc., Winooski, VT, United States).

After determining the total biomass and sugar release, the theoretical ethanol yield was calculated considering the total biomass conversion per surface area unit (ha) according to the National Renewable Energy Laboratory Standards (Dowe and McMillan, 2008), as follows [1]:

Theoreticalethanolyield(L/ha)=[Sugarrelease(ul/mg)x0.511x Biomass(kg/ha)]/1000[1]

In addition, other data were also accounted for throughout the experiment: (1) the plant heights on the same days on which the UAV flights were performed, (2) starting and ending dates of plant anthesis, as well as dates of plant and spikes emergences, (3) damages due to pests and diseases, specifically stem rust and barley yellow dwarf (BYD) virus, as well as ones provoked by birds and rodents.

### Data Analysis

The field trial design was generated with the statistical software R version 3.3.1 (R Core Team, 2016) and its function design.rcbd (Mendiburu, 2016), and data analysis was conducted with the statistical software JMP version 10 software (SAS Institute, Inc., Cary, NC, United States). First, the variability of plant phenotypic data was studied by using analysis of the variance (ANOVA). Next, capability of the UAV-based phenotyping system to predict bioethanol potential was studied by analyzing the degree of correlation (in terms of the coefficient of determination, *R*^2^) of multi-temporal UAV-based VIs with total biomass dry weight, sugar release, and theoretical ethanol yield. These correlations were determined in several TSs, as follows: (1) on each single date of the seven UAV flights (TS-1), (2) averaging the values of each VI obtained during plant anthesis (TS-2), and (3) averaging the values of each VI obtained during the full crop development (TS-3). TS-2 was proposed because anthesis is a critical period for cereal grain filling, which could also have an influence on the plant biomass accumulation and sugar left in the stems (Yang and Zhang, 2006; Barmeier and Schmidhalter, 2017) and, hypothetically, increase the spectral differences between the studied accessions. Finally, the VI and TS that reported the highest coefficient of determination were adjusted to a lineal model, which allowed the plant accessions to be ranked in terms of theoretical ethanol yield.

## Results

### Variability of Plant Phenotypic Data

**FIGURE 4:**
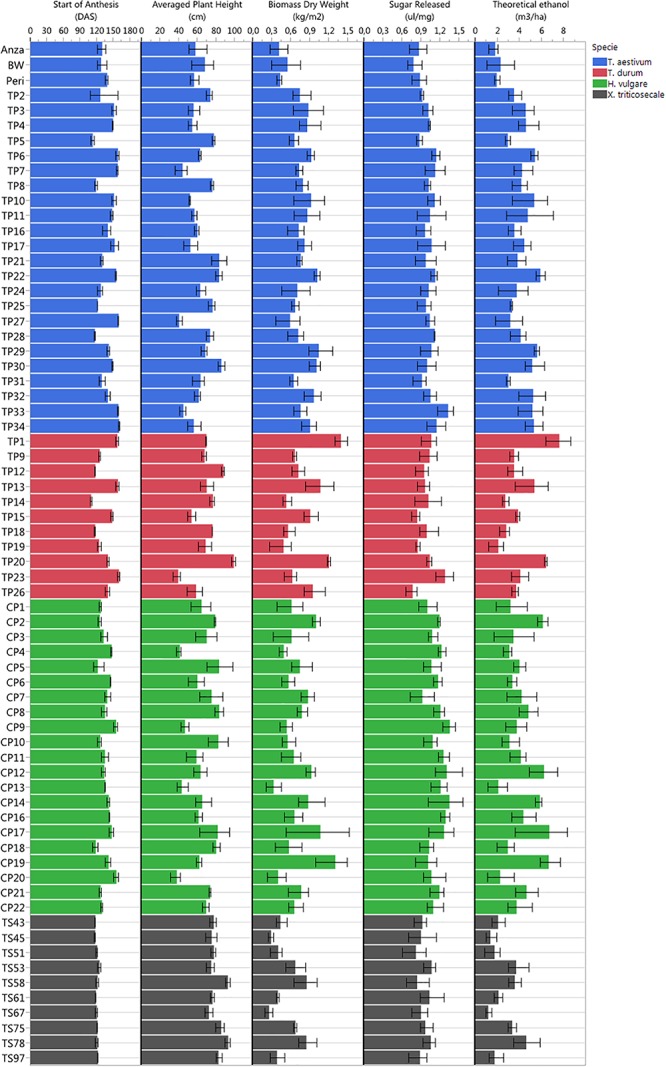
Plant phenotyping variability observed in the study trial plots in terms of start of plant anthesis (DAS), plant height, total biomass dry weight, sugar release, and theoretical ethanol yield.

All cultivars were well-adapted to Mediterranean climate conditions, which were favorable due to the low incidence of pests and diseases during the growing season in the studied campaign. The studied varieties were hardly affected by stem rust or BYD virus, and only occasional bird attacks of moderate importance were accounted for. Given the multitude of screened genotypes, many different phenotypes were observed in the study trial plots in terms of plant anthesis, plant heights, total biomass dry weight, sugar release and theoretical ethanol yield (Figure 4), which suggested high potential for ranking the observed phenotypes.

The earliest plant anthesis started for *T. durum* TP14 (Jupateco 73S) and *T. aestivum* TP5 (Opata 85) accessions at 110 and 112 DAS, respectively, and the latest plant anthesis started 160 DAS for *T. durum* TP23 (Avalon), *T. aestivum* TP34 (Cayuga) and *T. aestivum* TP27 (Renan) accessions. The early anthesis date of some accessions could be due to a short duration of their vegetative stage (Jamieson et al., 1998), which might have produced a smaller number of leaf primordia and resulted in a lower sink capacity and a decrease in biomass accumulation during the pre-anthesis period (Giunta et al., 1999). This was partially observed at the level of cereal species, in which the average dates of anthesis and the total biomass for *x Triticosecale* were significantly lower in comparison to the average values for other three species that were studied.

Plant height was a highly variable factor among genotypes over time. The average plant heights collected in the field during crop development ranked from minimum values of 38–40 cm for *H. vulgare* CP20 (Franka), *T. durum* TP23 (Avalon) and *T. aestivum* TP27 (Renan) accessions, to maximum values of 92–99 cm for *x Triticosecale* TS58 (Wapiti) and TS78 (Juanillo 95) and for *T. durum* TP20 (Rugby) accessions. At the level of cereal species, the average heights for *T. aestivum* and *x Triticosecale* (62.24 and 80.93 cm, respectively) were significantly smaller and larger, respectively, than for other species; however, *H. vulgare* and *T. durum* did not show significant differences in the average plant heights (65.90 and 69.87 cm, respectively).

Regarding the bioethanol-related variables, the values of total biomass ranked from 0.26 to 0.29 kg/m^2^ measured for *x Triticosecale* TS67 (Navojoa) and TS45 (Zebra) accessions, respectively, to 1.31–1.40 kg/m^2^ measured for *H. vulgare* CP19 (Franklin) and *T. durum* TP1 (IDO444) accessions, respectively; the values of sugar release ranked from 0.77 ul/mg measured for *T. durum* TP26 (Cayuga) and BW (Bobwhite) accessions to 1.35–1.36 ul/mg measured for *H. vulgare* CP9 (Steptoe) and CP14 (Oregon wolfe barley recessive) accessions, respectively; and the values of theoretical ethanol yield ranked from 1.18 m^3^/ha for *x Triticosecale* TS67 (Navojoa) accession to 7.60 m^3^/ha for *T. durum* TP1 (IDO444) accessions.

### Variability of the Vegetation Index (VI) Values Over Time

**FIGURE 5:**
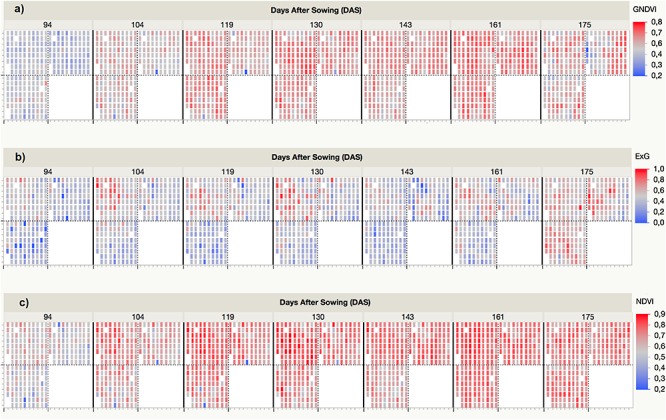
Spectral variability of three selected vegetation indices (VIs) measured by the UAV-based phenotyping system over time: **(a)** GNDVI, **(b)** ExG, **(c)** NDVI.

The VI values were automatically retrieved from the multi-spectral images collected at each UAV flight and by applying the customized OBIA procedure that was developed in this investigation. As an example, Figure 5 shows the progress of the three VIs (i.e., ExG, NDVI, and GNDVI) that produced better results in predicting some of the bioethanol-related variables. The ExG values varied from a minimum of 0.14 for *T. aestivum* Anza measured at the first date (94 DAS) to a maximum of 0.73 for *H. vulgare* CP7 (L94) and CP14 (OWB recessive) at the last date (175 DAS). The normalized difference vegetation index (NDVI) values varied from a minimum of 0.36 for *T. aestivum* BW (BobWhite) measured at the first date (94 DAS) to a maximum of 0.84 for *H. vulgare* CP8 (Fredrickson) and CP11 (Lina) at the fourth date (130 DAS). The GNDVI values varied from a minimum of 0.38 for *T. aestivum* BW (BobWhite) measured at the first date (94 DAS) and *H. vulgare* CP5 (Mokusekko 3) and *x Triticosecale* TS67 (Navojoa) at the last date (175 DAS) to a maximum value of 0.72 for *T. aestivum* TP30 (Thatcher) at the 6^th^ date (161 DAS).

**FIGURE 6:**
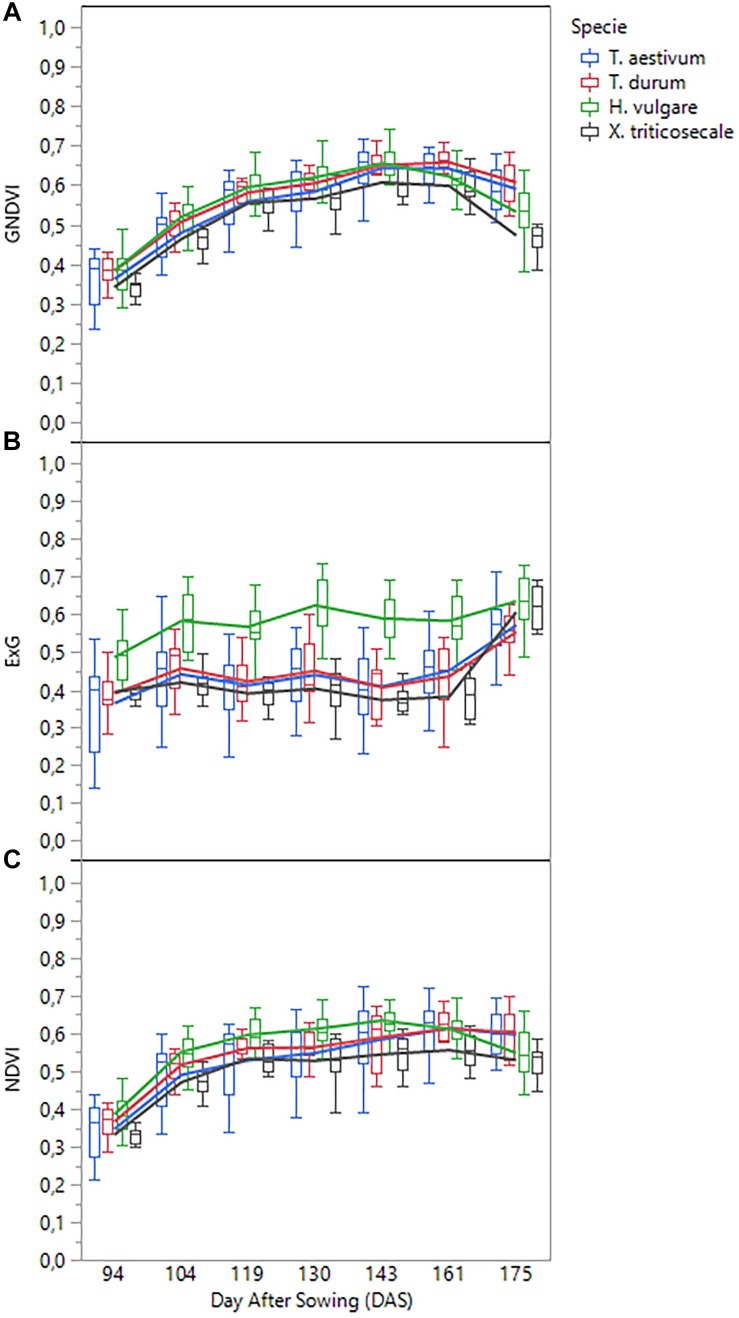
Temporal profile of three selected vegetation indices as affected by cereal species: **(A)** GNDVI, **(B)** ExG, **(C)** NDVI.

A detailed analysis of the variation of each VI over time confirmed that two different spectral patterns were observed (Figure 6). On the one hand, visible-based VIs (i.e., ExG, VIgreen, and TCI) generally followed a horizontal trend, characterized by minor variations from date 1 (94 DAS) to date 6 (161 DAS), but with a marked increase in the last date (175 DAS); the increase was most pronounced for species *T. aestivum*, *T. durum*, and *x Triticosecale*. The spectral profiles for these three species was analogous in the three visible-based VIs studied, although the mean values for two *Triticum* species were slightly higher than those for *x Triticosecale* species between the date 2 (104 DAS) and 6 (161 DAS), but all three species showed significantly lower values than those for *H. vulgare* species on all the studied dates. On the other hand, the values of NIR-based VIs (i.e., NDVI, GNDVI, MSR, and MCARI) generally described a bell-shaped curve. Depending on the accession considered, the maximum values were mostly around the 4^th^ (130 DAS), 5^th^ (143 DAS) or 6^th^ (161 DAS) dates, while the values of the previous dates progressed increasingly, and those of the following dates declined from the maximum; some accessions even reaching values on the last date that were close to those that were obtained during the earliest dates of the study. In this case, the lowest average values were always for the *x Triticosecale* species for all dates studied, while the highest values were observed for the *H. vulgare* species up to the 5^th^ date (143 DAS) for GNDVI and MSR and up to the 6^th^ date (161 DAS) for NDVI and MCARI. From these dates onward, the maximum average values of these four NIR-based VIs were reached for *T. durum* species followed by *T. aestivum* species. This turning point corresponded to cereal anthesis in most species, which suggested that special attention should be given to the UAV images that were taken during the anthesis period.

### Performance of UAV-Based Vegetation Indices to Predict Bioethanol-Related Variables

Simple linear regression analysis showed the degree of correlation (in terms of coefficient of determination) between VIs and crop total biomass dry weight, sugar release and theoretical ethanol yield (Table 3), which may indicate the ability of the UAV-based system to predict the bioethanol potential of the studied cereals. Of the seven dates studied in our experiment, we observed that the correlations were generally higher during the anthesis of each accession; hence, our proposal to study the three TSs described in Section “Data Analysis.” For the interpretation of the results, correlations were considered hereafter as low (*R*^2^ < 0.50), moderate (0.50 ≤ *R*^2^ < 0.60) and high (*R*^2^ ≥ 0.60).

**TABLE 3:** The coefficient of determination (*R*^2^) of the linear relationship between the studied vegetation indices and crop total biomass dry weight, sugar release, and theoretical ethanol yield as affected by the three temporal scenarios (TSs) studied.

	Single dates, in DAS (TS-1)^*^							Combined dates^*^	
**Bioethanol-related variable/vegetation index**	**94**	**104**	**119**	**130**	**143**	**161**	**175**	**On anthesis (TS-2)**	**Full crop development (TS-3)**
**Total biomass**									
Visible									
ExG	0.18^*^	0.22^*^	0.16^*^	0.15^*^	0.11			0.16^*^	0.17
VIgreen	0.19^*^	0.23^*^	0.16^*^	0.19^*^	0.14^*^	0.27^*^		0.22^*^	0.23^*^
TCI	0.29^*^	0.30^*^	0.25^*^	0.17^*^	0.21^*^	0.15	0.33^*^	0.19	0.37^*^
**Visible and NIR**									
NDVI	0.41^*^	0.41^*^	0.40^*^	0.35^*^	0.45^*^	0.50^*^	0.34^*^	0.54^*^	0.58^*^
GNDVI	0.40^*^	0.40^*^	0.40^*^	0.39^*^	0.49^*^	0.51^*^	0.35^*^	0.57^*^	**0.59** ^*^
MCARI	0.39^*^	0.39^*^	0.41^*^	0.39^*^	0.47^*^	0.48^*^	0.40^*^	0.41^*^	0.58^*^
MSR	0.30^*^	0.32^*^	0.37^*^	0.35^*^	0.40^*^	0.41^*^	0.41^*^	0.52^*^	0.56^*^
**Sugar release**									
Visible									
ExG	0.29^*^	0.43^*^	0.45^*^	0.48^*^	0.47^*^	0.39^*^		**0.57** ^*^	0.51^*^
VIgreen	0.28^*^	0.42^*^	0.43^*^	0.46^*^	0.46^*^	0.36^*^		0.52^*^	0.48^*^
TCI	0.18^*^	0.44^*^	0.47^*^	0.43^*^	0.39^*^	0.32^*^	0.12	0.52^*^	0.48^*^
**Visible and NIR**									
NDVI	0.18^*^	0.41^*^	0.42^*^	0.45^*^	0.45^*^	0.24^*^		0.34^*^	0.44^*^
GNDVI	0.17^*^	0.28^*^	0.26^*^	0.26^*^	0.25^*^			0.20	0.23^*^
MCARI	0.16^*^	0.35^*^	0.33^*^	0.37^*^	0.45^*^	0.17^*^		0.37^*^	0.39^*^
MSR	0.13	0.29^*^	0.31^*^	0.32^*^	0.45^*^	0.22^*^		0.25^*^	0.39^*^
**Theoretical ethanol yield**									
Visible									
ExG	0.26^*^	0.35^*^	0.28^*^	0.27^*^	0.24^*^	0.21^*^		0.32^*^	0.32^*^
VIgreen	0.27^*^	0.36^*^	0.28^*^	0.32^*^	0.27^*^	0.39^*^		0.37^*^	0.37^*^
TCI	0.36^*^	0.42^*^	0.37^*^	0.27^*^	0.32^*^	0.27^*^	0.33^*^	0.34^*^	0.51^*^
**Visible and NIR**									
NDVI	0.46^*^	0.51^*^	0.48^*^	0.46^*^	0.54^*^	0.55^*^	0.34^*^	0.62^*^	**0.66** ^*^
GNDVI	0.43^*^	0.47^*^	0.45^*^	0.45^*^	0.53^*^	0.45^*^	0.28^*^	0.58^*^	0.61^*^
MCARI	0.45^*^	0.50^*^	0.50^*^	0.48^*^	0.54^*^	0.49^*^	0.35^*^	0.53^*^	0.65^*^
MSR	0.37^*^	0.42^*^	0.48^*^	0.44^*^	0.52^*^	0.48^*^	0.37^*^	0.57^*^	0.65^*^

Significant *R*^2^ values at *p* ≤ 0.001 are marked with an asterisk, while insignificant *R*^2^ values are not shown. White, gray, and black cells indicate low (*R*^2^ < 0.50), moderate (0.50 ≤ *R*^2^ < 0.60) and high (*R*^2^ ≥ 0.60) correlations, respectively. The bold numbers and the underlined numbers enphasize the best result of each VI and each TS, respectively. ^*^Dataset: 234 samples for each date of TS-1, 495 samples for the combined dates of TS-2, and 1638 samples for the combined dates of TS-3.

NIR-based VIs showed better correlation with the crop total biomass dry weight than visible-based VIs for all dates and TS considered, although no high *R*^2^ values were obtained in any case. On single dates (TS-1), moderate correlations were found only on the 6^th^ date (161 DAS), with *R*^2^ values of 0.50 and 0.51 for NDVI and GNDVI, respectively, while correlations on other dates were low. The NDVI and GNDVI reported slightly better results in the TS-2 (i.e., averaged VI values during plant anthesis), with *R*^2^ values of 0.54 and 0.57, respectively, and they were even better in the TS-3 (i.e., averaged VI values during full crop development), with *R*^2^ values of 0.58 and 0.59, respectively. Moderate correlations were also obtained with MSR in the TS-2 and TS-3 (with *R*^2^ values of 0.52 and 0.56, respectively), and with MCARI in TS-3 (*R*^2^ of 0.58). Regarding visible-based VIs, correlations were low in all cases, with maximum values of 0.33 and 0.37 obtained by the TCI on the last date (175 DAS) and in TS-3, respectively.

However, the results differed when studying the linear relationship of VIs to sugar release. In this case, the highest correlations were obtained with visible-based VIs in all the TS considered, with moderate *R*^2^ values ranging from 0.52 (VIgreen and TCI) to 0.57 (ExG) in the TS-2, and with a *R*^2^ value of 0.51 obtained for ExG in the TS-3. For the rest of the cases, the correlations were low, although visible-based VIs obtained higher *R*^2^ values than NIR-based VIs on all the single dates studied, in particular ExG on the 4^th^ (130 DAS) and 5^th^ (143 DAS) dates (*R*^2^ of 0.48 and 0.47, respectively) and TCI on the 2^nd^ (104 DAS) and 3^rd^ (119 DAS) dates (*R*^2^ of 0.44 and 0.47, respectively).

Regarding the prediction of theoretical ethanol yield, great correlations were obtained with the four NIR-based VIs in TS-3, with *R*^2^ values between 0.61 (GNDVI) and 0.66 (NDVI). The NDVI also obtained a high correlation with theoretical ethanol yield in TS-2 (*R*^2^ of 0.62), while better correlations for singles dates were obtained with NDVI on the 6^th^ (161 DAS) date (*R*^2^ of 0.55) and with the four NIR-based indices on the 5^th^ (143 DAS) date (*R*^2^ ranged from 0.52 to 0.54). In constrast, visible-based indices obtained low correlations in almost all the TS studied except for TCI, which obtained a moderate correlation (*R*^2^ of 0.51) with theoretical ethanol yield in TS-3.

At the level of cereal species, ordering and significant values reported for the selected VIs and the three bioethanol-related variables were equal (Table 4). *T. durum* had significantly higher average values than those of the other screened species for GNDVI (0.57) and total biomass (0.83 kg/m^2^), while *H. vulgare* was significantly higher for ExG (0.59) and sugar release (1.16 ul/mg). Both species obtained significantly higher mean values of NDVI (0.54 and 0.56, respectively) and theoretical ethanol yield (4.14 and 4.21 m^3^/ha, respectively) than *T. aestivum* and *x Triticosecale*. In all the factors, *x Triticosecale* showed significant lowest mean values compared to that of the other species, which confirmed the good performance of the selected VIs in predicting a ranking of cereal species in terms of bioethanol potential.

**TABLE 4:** Mean values and ANOVA of the GNDVI, ExG, and NDVI indices selected at the best temporal scenario (TS) for phenotyping of total biomass, sugar release and theoretical ethanol yield, respectively, at the level of cereal species.

	**GNDVI (in TS-3) vs. total biomass dry weight**		**ExG (in TS-2) vs. sugar release**		**NDVI (in TS-3) vs. theoretical ethanol yield**	
**Cereal species**	**GNDVI**	**Biomass (kg/m^2^)**	**EXG**	**Sugar release (ul/mg)**	**NDVI**	**Theoretical ethanol yield (m^3^/ha)**
*T. aestivum*	0.54 b	0.67 b	0.44 b	0.98 b	0.52 b	3.49 b
*T. durum*	0.57 a	0.83 a	0.43 b	0.98 b	0.54 ab	4.14 a
*H. vulgare*	0.55 b	0.71 b	0.59 a	1.16 a	0.56 a	4.21 a
*x Triticosecale*	0.51 c	0.52 c	0.38 c	0.94 c	0.49 c	2.54 c

*Within a column mean values followed by the same letter do not differ significantly according to least significant difference (LSD) test at *P* = 0.05.*

## Discussion

Several investigations have recently demonstrated the capability of UAVs for collecting phenotypic data on numerous crops and case studies (Sankaran et al., 2015b; Shi et al., 2016; Yang et al., 2017). This study went beyond this by presenting the first experiment with a UAV-based multi-spectral system for phenotyping several characters of a population of known genotypes of wheat, barley, and triticale with the purpose of identifying the best accessions for bioethanol production. The field trial showed high variability in plant height, anthesis dates, and bioethanol-related factors such as total dry biomass, sugar release, and theoretical ethanol yield. Variability was not significantly associated with grain yields (Ostos-Garrido et al., 2018), which was consistent with previous investigations in barley (Capper, 1988) and wheat (Jensen et al., 2011; Lindedam et al., 2012). This is a key aspect in breeding programs that aim to select plants with better straw quality for bioethanol production but without sacrificing grain yield.

Since there was phenotypic variability in the experiment, the challenge was to quantify this variability with an efficient and reliable system. The UAV-based phenotyping system first computed the spectral variability of plant material (234 cereal plots) over time, and then the system estimated the bioethanol-related variables with acceptable precision by using selected image-based vegetation indices calculated at specific temporal intervals. At this point, it is relevant to highlight the influence of the temporal factor to achieving better estimations. As can be observed in Table 3, the results of TS-1 (i.e., on each single date) were always lower than those obtained in TS-2 (i.e., averaging the values of each VI obtained during plant anthesis) and TS-3 (i.e., averaging the values of each VI obtained during full crop development). This result suggests that future research on crop phenotyping should include a multi-temporal study, and in some cases, should primarily consider the crop anthesis dates (TS-2). For example, predictions of sugar release in our experiment were more accurate with visible-based VIs that were calculated in TS-2 (*R*^2^ from 0.52 to 0.57) than in TS-3 (*R*^2^ from 0.48 to 0.51).

A general result was that NIR-based VIs (i.e., NDVI, GNDVI, MCARI, and MSR in this study) were more appropriate to estimate crop biomass, meanwhile visible-based VIs (i.e., ExG, VIgreen, and TCI in this study) were suitable for crop sugar release. Estimations were low on most of the single dates that were studied (TS-1), although moderate correlations between the NDVI and GNDVI with the crop biomass were obtained on DAS 161 (*R*^2^ of 0.50 and 0.51, respectively). The difference between both VIs is that NDVI uses NIR-red and GNDVI uses NIR-green spectral bands. Therefore, it seems that the green band, which is sensitive to small changes in the vegetation greenness and canopy, was slightly more correlated to the crop biomass. However, better biomass estimations were obtained when GNDVI was calculated during plant anthesis (TS-2, *R*^2^ of 0.57), and even better estimations were obtained when NDVI, GNDVI, and MCARI were calculated during full crop development (TS-3, *R*^2^ of 0.58–0.59).

In the case of sugar release, EXG calculated during plant anthesis (TS-2) revealed the highest correlation (*R*^2^ of 0.57) of the studied VIs. To our knowledge, there are no previous studies that explain the indirect relationship that may exist between a visible-based index and sugar release. Lindedam et al. (2010) and Bekiaris et al. (2015) revealed some near- and mid- infrared spectral regions with a significant contribution in the prediction of the total sugar release by applying spectroscopy analysis and they partially attributed their results to plant senescence components such as lignin, cellulose, and hemicellulose. However, our experiment reported the best estimations prior to crop senescence, which point to components or aspects related to green vegetation. Upward progress of ExG correlations to reach a maximum during crop anthesis may be explained by the influence that changes of crop greenness and plant pigments have on ExG measurements (Yu et al., 2013; Torres-Sánchez et al., 2014). Plants have the greatest amount of carbon in the form of sugars and the maximum number of leaves during anthesis, which are easily degradable plant material (Jensen et al., 2011; Lindedam et al., 2012; Bellucci et al., 2015). ExG correlations decreased after crop anthesis (i.e., from 161 DAS onwards in almost all accessions), just as the plants progressed toward senescent stages caused by the decrease in the proportion of chlorophyll (green pigments) in favor of anthocyanin (red pigments).

**FIGURE 7:**
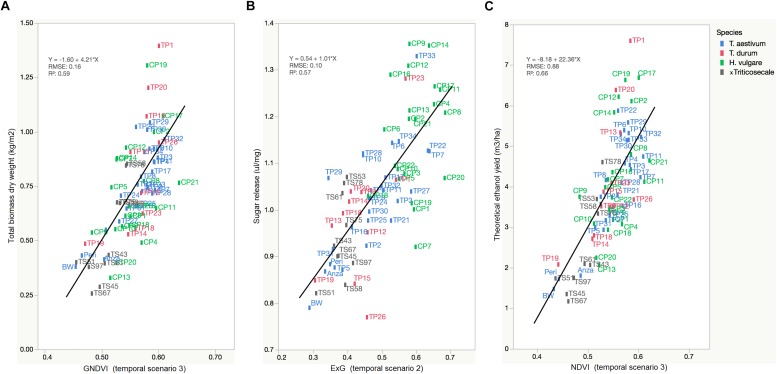
Linear regressions of selected VIs against the crop variables that are particularly related to bioethanol potential showing the rank of cereal accessions: **(A)** GNDVI (in TS-3) vs. Total biomass dry weight (kg/m^2^), **(B)** ExG (in TS-2) vs. Sugar release (ul/mg), **(C)** NDVI (in TS-3) vs. theoretical ethanol yield (m^3^/ha).

The primary purpose of phenotyping techniques is to provide a ranking of the studied plants to facilitate or accelerate the process of selecting genetic material for the next stages of plant breeding. However, a ranking is not provided in many cases, which reduces the impact of the experiment to monitoring only one or various crop variables. Our UAV-based system aimed to rank the cereal accessions in terms of theoretical ethanol yield as a result of the linear combination of the total biomass and sugar release (see Eq. 1). In this case, the linear equation of GNDVI, ExG, and NDVI served to predict a ranking of accessions for each crop variable related to bioethanol potential (Figure 7).

The range of selected VI values ordered the large variability of cereal accessions with a root mean square error (RMSE) of prediction of 0.16 kg/m^2^ for total biomass, 0.10 ul/mg for sugar release, and 0.88 m^3^/ha for theoretical ethanol yield. This ranking showed the cereal accessions with higher and lower values of each variable and, consequently, demonstrated the value of the UAV-based system for the early and non-destructive identification of improved varieties that combine high saccharification potential and high biomass production for a cereal breeding program. The UAV-based system mostly pointed out *H. vulgare* accessions [e.g., CP21 (Azumamugi), CP17 (Cebada Capa), CP12 (Apex), CP19 (Franklin), among others] as being those with the best lignocellulosic source for bioethanol production, followed by *Triticum* accessions [e.g., TP11 (P92201), TP32 (USG 3209), and TP1 (IDO444)] and, finally, *x Triticosecale* accessions, which generally agreed with the results of the laboratory analysis of cereal straw (Table 4). These results were also in line with the previous research of Chen et al. (2007a, b), which evaluated the production of bioethanol from several straws and hays, and highlighted barley as a major source of biomass and having a greater potential for bioethanol than the other cereals studied.

Some of the contrasting accessions observed in the ranking, e.g., CP9 (Steptoe) × CP10 (Morex) and OWB (CP13 and CP14) have been used as parental lines in mapping populations for different characters (Kleinhofs et al., 1993), including regulatory genes (Tondelli et al., 2006), resistance to rust (Arru et al., 2003), and to develop a genetic link map of SNP by consensus in barley (Close et al., 2009), which is an important resource for genetic studies. As a first approximation, our results suggest that some barley mapping populations, e.g., CP13 (OWB dominant) × CP14 (OWB recessive) and CP19 (Franklin) × CP13, could be good candidates for identifying the genetic factors underlying the difference in theoretical bioethanol potential and in wall recalcitrance.

## Conclusion

This study demonstrated the capability of an UAV-based multi-spectral images to rank several accessions of wheat, barley, and triticale in terms of their potential for bioethanol production with satisfactory accuracy. Analysis of the multi-spectral data shown that the GNDVI, ExG, and NDVI correlated well with total biomass dry weight, sugar release and theoretical ethanol yield, respectively, and in all cases, the temporal component was fundamental. In the case of biomass and ethanol yield, the best result was obtained by averaging the values of GNDVI and NDVI obtained during full crop development, while in the case of sugar it was averaging the values of ExG obtained during plant anthesis.

While genotypic tools have greatly advanced technologically, phenotypic tools remain the main bottleneck in the decision-making process. However, the innovative UAV-based phenotyping system described in this article can enrich breeding programs for bioethanol production by drastically accelerating the timing to capture and process the field trial data. In fact, the time required to conduct the entire process, from operating the UAV flight to computing the spectral data, was approximately 1 h, while obtaining phenotypic data from 234 cereal plots by using conventional laboratory techniques took several days. Therefore, although the UAV-based predictions were moderate in most cases, the potential to save time and resources certainly justifies the usefulness of this technology. Additionally, this research reported the phenological dates and specific spectral regions (i.e., vegetation indices) that provide reliable beforehand information to predict the biomass and sugar content of the studied plants, whose large variability constituted a valuable resource for cereal genetics studies.

## Author Contributions

FP and JP conceived and designed the experiments, and contributed with equipment and analysis tools. FO-G and FP performed the cereal field experiments. JT-S and JP performed the UAV flight experiments. FO-G, AdC, and JP analyzed the data and wrote the manuscript. All authors have read and approved the manuscript.

## Conflict of Interest Statement

The authors declare that the research was conducted in the absence of any commercial or financial relationships that could be construed as a potential conflict of interest.

## Footnotes

1 http://www.ars.usda.gov/

2 http://earth.nig.ac.jp/~dclust/cgi-bin/index.cgi?lang=en
